# Dr. George C. McNett

**Published:** 1909-12

**Authors:** 


Dr. George C. McNett, of Bath, N. Y., died at his home in that village, October 15, 1909, aged 52 years. He was the son of General A. J. McNett, and was born in Buffalo, July 11, 1857. His father was a prominent member of the Buffalo bar and had distinguished himself in the Civil War. Dr. McNett's early education was obtained in schools at Belmont, N. Y., at Saint Joseph's College, Buffalo, and at Alfred University. He took his medical degree at the University of New York Medical College in 1881, and began practice at Belmont. In 1886, he was appointed surgeon at the State Home for Soldiers and Sailors at Bath. He resigned in 1890 to engage in general practice in Bath.

He was married in early life to Agnes, daughter of E. S. S. Stewart, of Belmont. Mrs. McNett died several years ago. A daughter, Miss Celia, alone survives.

